# Clinical Management and Molecular Landscape of Combined Hepatocellular–Cholangiocarcinoma: A Comprehensive Review

**DOI:** 10.3390/jcm15218429

**Published:** 2026-10-29

**Authors:** Lo-Yip Yu, Hung-Ju Ko, Yang-Che Kuo, Ying-Chun Lin, Shou-Chuan Shih, Chuan-Chuan Liu, Kuang-Chun Hu

**Affiliations:** 1 Healthy Evaluation Center, Mackay Memorial Hospital, Taipei 10449, Taiwan; benny.6285@mmh.org.tw (L.-Y.Y.); bonnie@mmh.org.tw (H.-J.K.); yangche@mmh.org.tw (Y.-C.K.); sschuan@mmh.org.tw (S.-C.S.); carrie@mmh.org.tw (C.-C.L.); 2 Division of Gastroenterology, Department of Internal Medicine, Mackay Memorial Hospital, Taipei 10449, Taiwan; 3 Department of Medicine, MacKay Medical University, Taipei 104, Taiwan; 4 Department of Anesthesiology, Mackay Memorial Hospital, Taipei 10449, Taiwan; anes.4911@mmh.org.tw; 5 Graduate Institute of Epidemiology and Preventive Medicine, College of Public Health, National Taiwan University, Taipei 104, Taiwan; 6 MacKay Junior College of Medicine, Nursing, and Management, Taipei 104, Taiwan

**Keywords:** combined hepatocellular–cholangiocarcinoma, biphenotypic liver cancer, hepatocellular carcinoma, intrahepatic cholangiocarcinoma

## Abstract

Combined hepatocellular–cholangiocarcinoma (cHCC-CCA) is a rare and highly aggressive primary liver cancer. It is distinguished by the unequivocal coexistence of both hepatocellular carcinoma (HCC) and intrahepatic cholangiocarcinoma (iCCA) components within a single tumor. cHCC-CCA accounts for between 0.4% and 14.2% of primary liver cancers, presenting a complex clinical and pathological challenge because of its dual histological morphology and phenotypic diversity. In the past, its classification faced significant diagnostic challenges. However, these issues have been largely addressed by the 2019 World Health Organization 5th Edition reclassification, which requires clear differentiation of hepatocytic and cholangiocytic features using hematoxylin and eosin (H&E) staining. Recent genomic profiling has shown that cHCC-CCA exists on a molecular spectrum, with about 75% of individual tumors being HCC-like or CCA-like. Biliary-dominant subtypes, similar to CCA, often contain actionable alterations like FGFR2 fusions, IDH1 mutations, and HER2 amplification, providing significant opportunities for precision oncology. In contrast, subtypes resembling HCC are commonly driven by mutations in TP53 and the TERT promoter. Major hepatectomy combined with regional lymphadenectomy continues to be a fundamental curative treatment. However, liver transplantation is increasingly being recognized as a viable option for carefully selected patients with early-stage cirrhosis. In the advanced or recurrent stage, the traditional preferred treatment continues to be cytotoxic gemcitabine combined with platinum-based therapies. Meanwhile, combinations involving immune checkpoint inhibitors, like atezolizumab and bevacizumab, or more complex regimens that integrate locoregional therapy, TKIs, and PD-(L)1 inhibitors, offer hopeful prospects for improving survival rates. This review provided an overview of the current knowledge of the clinical classification, risk stratification, histogenesis, molecular biology and clinical management of cHCC-CCA to inform current management approaches and outline future paths in precision oncology.

## 1. Introduction

Primary liver cancer is one of the most formidable oncology challenges globally. It is the sixth most frequently diagnosed cancer and the third leading cause of cancer-related deaths worldwide [1]. Most primary hepatic epithelial malignancies predominantly include either hepatocellular carcinoma (HCC), which arises from hepatocytes, or intrahepatic cholangiocarcinoma (iCCA), which develops from the epithelial lining of the intrahepatic bile ducts. A rare and highly aggressive hybrid variant exists [2], known as combined hepatocellular–cholangiocarcinoma (cHCC-CCA); this distinct malignancy exhibits an unequivocal mixture of both hepatocytic and cholangiocytic differentiation lineages within the same tumor nodule [2]. Its distinctive biphenotypic nature leads to an aggressive clinical progression, often resulting in prognostic outcomes that are typically worse than those of pure HCC and comparable to or even more severe than pure iCCA [3].

cHCC-CCA is considered a rare tumor, with incidence rates reportedly ranging from 0.4% to 14.2% of all primary liver cancers [4]. Large population-based retrospective database studies, such as those extracted from the United States Surveillance, Epidemiology, and End Results (SEER) program, suggest an annual incidence of approximately 0.05 per 100,000 individuals. This figure has been increasing, as have mortality rates, largely due to advancements in pathological screening and more precise staging methods observed over recent decades [5]. While historical data may have underestimated the true prevalence of cHCC-CCA due to sampling limitations in small core biopsies and diagnostic confusion, modern pathology series show that it represents 2% to 5% of all primary liver cancers [6]. The median age of diagnosis is between 50 and 75 years, with the peak incidence typically observed in the sixth and seventh decades of life [7].

This review provided an overview of the current knowledge of the clinical classification, risk stratification, histogenesis, molecular biology and clinical management of cHCC-CCA. As we deepen our understanding of the pathogenesis of cHCC-CCA, particularly its molecular biological and immunological aspects, it is anticipated that more immunotherapy and chemotherapy options will become available in the future, benefiting a greater number of patients.

## 2. Historical Evolution of Classification Systems for cHCC-CCA

The history of cHCC-CCA is marked by significant terminological confusion and classification updates, reflecting the evolving scientific understanding of its pathogenesis.

The disease was first described by Wells in 1903 as a primary liver carcinoma demonstrating dual differentiation [8]. In 1949, Allen and Lisa proposed the first systematic classification system based on five resected cases, dividing the disease into three distinct categories: Type A (double tumor), characterized by separate, distinct nodules of HCC and iCCA; Type B (combined type), exhibiting contiguous nodules with zones of intermingling; and Type C (mixed type), defined by a single mass showing an intimate, fine-scale intermixture of both malignant components [9].

In 1985, Goodman and colleagues modified this framework into Type I (collision tumor, corresponding to Allen Type B), Type II (transitional tumor, corresponding to Allen Types A and C, characterized by progressive transition and intermediate differentiation), and Type III (fibrolamellar tumor, resembling fibrolamellar HCC but containing mucin-producing pseudo-glands) [10].

The World Health Organization (WHO) first codified cHCC-CCA as a distinct, independent disease entity in its 2010 (4th Edition) classification of tumors of the digestive system. This edition divided cHCC-CCA into the ‘classical type’ and ‘subtypes with stem cell features’ including typical, intermediate-cell, and cholangiocellular variants, based on immunohistochemical and morphological stem cell marker expressions [11]. This classification encountered considerable reproducibility challenges among pathologists, because stem cell markers are commonly expressed in various traditional liver malignancies [12].

To address these clinical challenges, an international consensus group in 2018 recommended a simplified framework, which was officially adopted in the 2019 (5th Edition) WHO classification [13]. Under the 5th Edition criteria, cHCC-CCA is strictly defined by the unequivocal coexistence of both hepatocytic and cholangiocytic components on routine hematoxylin and eosin (H&E) staining regardless of the percentage of each component [12]. True collision tumors, which develop independently, are not included. The cholangiocellular carcinoma subtype is now classified under small duct iCCA, and intermediate cell carcinoma is recognized as a unique, separate category [14]. Historical evolution of classification systems for cHCC-CCA are noted in Table 1.

## 3. Risk of cHCC-CCA

A striking feature of cHCC-CCA is its marked geographical heterogeneity, which greatly affects the distribution of risk factors, the biology of tumors, and the tolerance to treatment.

In East Asian cohorts, particularly in China, Japan, and South Korea, cHCC-CCA is highly prevalent in males, with a male-to-female ratio ranging from 2:1 to 8:1 [15].

In these populations, the main causes closely align with those of traditional HCC: chronic hepatitis B virus (HBV) infection, which accounts for more than 50% to 80% of cases, and underlying liver cirrhosis, found in 35% to 68% of patients [16]. Chronic hepatitis C virus (HCV) infection, excessive alcohol intake, and metabolic syndrome are considered secondary risk factors [16].

In contrast, Western populations exhibit a more balanced sex distribution, with male-to-female ratios ranging from 1:1 to 2:1, and a lower incidence of underlying cirrhosis, which often develops anew in non-cirrhotic liver tissue [17]. In the West, HCV infection, metabolic dysfunction-associated steatotic liver disease (MASLD), and alcoholic liver disease represent the dominant risk backgrounds [18].

Beyond general risk backgrounds, several specific risk associations are noteworthy. In direct contrast to conventional HCC, cHCC-CCA shares specific risk links with iCCA. The most definitive of these is an association with liver fluke infections, specifically Clonorchis sinensis and Schistosoma mansoni, which induce chronic biliary inflammation, bile duct hyperplasia, and eventual malignant transformation of hepatic progenitor cells [19]. Sporadic cases in the United States and the Philippines have reinforced this etiological link, though it remains a minor contributor compared to viral hepatitis [19]. Additionally, a significant clinical association has been observed between the emergence of mixed/biphenotypic HCC following trans-arterial chemoembolization (TACE) for an existing conventional HCC. This suggests that TACE-induced ischemic hypoxia and necrosis might lead to clonal selection or trans-differentiation of surviving cells into a biliary phenotype [20].

## 4. Clinical Presentation and Diagnostic Pathways

In its early stages, cHCC-CCA tends to progress quietly, often without noticeable symptoms. Most patients either remain symptom-free or experience vague signs like discomfort in the right upper quadrant, abdominal pain, fatigue, a slight fever, and weight loss [21]. Consequently, the majority of cases are diagnosed at an advanced or unresectable stage during routine screening for chronic liver disease or as an incidental finding [16]. A key feature of cHCC-CCA is its dual biological behavior. It often exhibits high rates of microvascular invasion, which can reach up to 68.7%. Like HCC, it frequently shows portal vein invasion and has a significant tendency for metastasizing to hilar lymph nodes, with rates ranging from 12% to 33%, and even up to 40% [22].

Preoperative diagnosis remains a significant clinical challenge due to the overlapping radiological and serum features. A diagnosis of cHCC-CCA may be strongly suspected when there is a clear discrepancy between serum tumor markers and imaging enhancement patterns [23]. The simultaneous rise in alpha fetoprotein (AFP), which is specific to HCC, alongside carbohydrate antigen 19-9 (CA19-9) or carcinoembryonic antigen (CEA), which are specific to iCCA, is noted in about 15% to 17.8% of cases, serving as a significant diagnostic indicator [24]. Furthermore, up to 51.1% to 53.5% of patients demonstrate a mismatch where a tumor exhibiting typical HCC-like enhancement on imaging is accompanied by elevated serum CA19-9 levels, or an iCCA-like enhancing mass is accompanied by elevated AFP levels [25]. However, the tumor markers including AFP and CA19-9 have different sensitivity and specificity with different serum cut-off threshold and disease stage, it must be interpreted with caution, as they can be elevated in benign biliary tract disorders or chronic hepatitis [23].

Imaging characteristics of cHCC-CCA on contrast enhanced ultrasound (CEUS) has been reported by Li et al. which demonstrated that 95.6% of the tumor showed washout enhancement pattern on CEUS, indicating a malignant nature of the tumor. Their results demonstrated that imaging features of cHCC-CCA may resemble either HCC or iCCA. HCC enhancement pattern and iCCA enhancement pattern are likely present in a comparable proportion in patients with cHCC-CCA on CEUS [25]. There is a certain correlation between the CEUS features and the presence of microvascular invasion in patients with cHCC-CCA. Low echo halo around the tumor, peripheral irregular rim-like enhancement of the nodules, and early washout are independent risk factors for microvascular invasion in cHCC-CCA patients, which have significant predictive value for the presence of microvascular invasion. It provides meaningful reference value for further treatment of patients [26].

On multi-phasic contrast-enhanced computed tomography and magnetic resonance imaging (gadoxetic acid-enhanced MRI), cHCC-CCA exhibits a mixed enhancement pattern reflecting its dual histological components [27]. The HCC-dominant component demonstrates typical arterial phase hyperenhancement followed by rapid washout on portal and delayed phases, along with an enhancing pseudo-capsule [18]. The iCCA-dominant component demonstrates a peripheral rim-like enhancement in the arterial phase followed by progressive, centripetal enhancement of the fibrous desmoplastic stroma on delayed phases, frequently associated with capsular retraction and localized biliary duct dilatation [18].

Most cHCC-CCA arise in the background of chronic hepatitis or cirrhosis; therefore, the primary differential imaging diagnosis is HCC. cHCC-CCAs showing typical imaging findings of HCC can be misdiagnosed and subjected to inappropriate treatment. Using only imaging modalities may lead to misdiagnosis of cHCC-CCAs. It is often subjected to biopsy for diagnosis of cHCC-CCA [28]. However, there may be sampling error, as only a part of the tumor tissue is sampled during biopsy. For appropriate pathologic diagnosis of cHCC-CCA, multiple site core biopsy or even surgical specimens including all tumor components are preferred [23].

**Figure 1:**
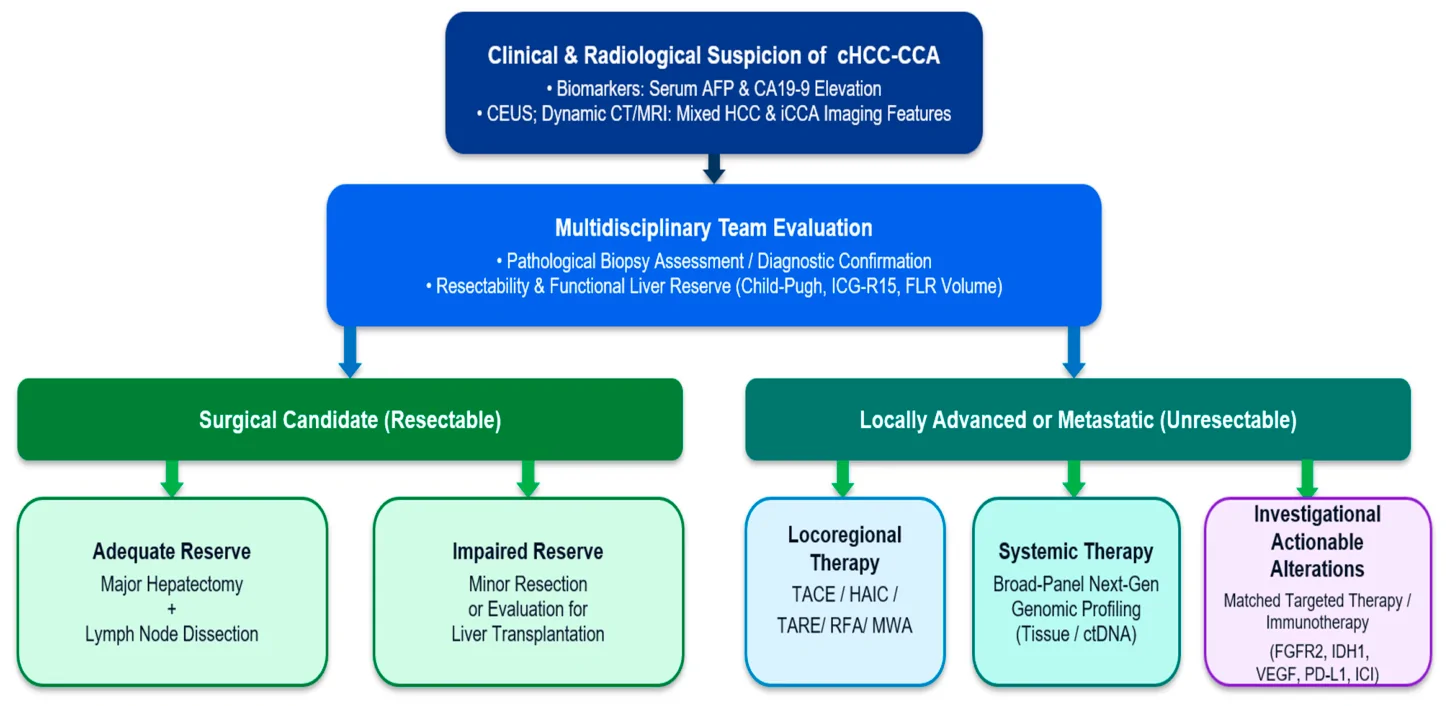
cHCC-CCA treatment decision flowchart. Abbreviations: cHCC-CCA—combined hepatocellular-cholangiocarcinoma, HCC—hepatocellular carcinoma, iCCA—intrahepatic cholangiocarcinoma, AFP—alpha-fetoprotein, CA19-9—carbohydrate antigen 19-9, CEUS—contrast enhanced ultrasound, CT—computed tomography, MRI—magnetic resonance imaging, ICG-R15—indocyanine green retention test at 15 minutes, FLR—future liver remnant, TACE—transcatheter arterial chemoembolization, TARE—transarterial radioembolization, HAIC—hepatic arterial infusion chemotherapy, RFA—radiofrequency ablation, MWA—microwave ablation, FGFR2—fibroblast growth factor receptor 2, IDH1—isocitrate dehydrogenase 1,VEGF—vascular endothelial growth factor, PD-L1—programmed cell death-ligand 1, ICI—immune checkpoint inhibitor, ctDNA—circulating tumor deoxyribonucleic acid.

Based on existing evidence and certain investigational treatment approaches—particularly in the fields of immunotherapy and targeted therapy—the potential clinical decision-making pathways are presented here in the form of a flowchart (Figure 1). Naturally, final treatment decisions should be reached through comprehensive consideration and discussion, taking into account the specific diagnostic tools and treatment modalities available at the patient’s hospital. The cHCC-CCA treatment decision flowchart is in Figure 1.

## 5. Histopathology and Pathological Diagnostics

Histological examination of resected surgical specimens is the gold standard for definitively diagnosing cHCC-CCA. This is because the significant spatial and histomorphological variability of these tumors often results in sampling errors and misdiagnosis when using small needle biopsies [29].

Morphologically, the classical type of cHCC-CCA is marked by the close intermingling or proximity of traditional HCC and iCCA regions. Intermediate transition zones illustrate a gradual morphological shift [29]. The HCC component is recognized by polygonal cells containing granular eosinophilic cytoplasm, prominent nucleoli, and bile or canalicular structures, arranged in trabecular, solid, or pseudo-glandular patterns embedded in a scant, delicate sinusoidal stroma [30]. Conversely, the iCCA component is composed of cuboidal or columnar mucin-producing epithelial cells forming true glandular, tubular, or acinar structures, surrounded by a dense, desmoplastic, collagen-rich fibrotic stroma [31].

Immunohistochemistry (IHC) is a crucial tool for supporting the confirmation of dual-lineage differentiation. This is particularly important in cases where histological differentiation is poor or in instances of intermediate cell carcinoma, where the morphology may be unclear [13]. To establish the hepatocytic lineage, pathologists utilize a highly sensitive and specific panel of markers: hepatocyte paraffin 1 (HepPar-1), arginase-1 (ARG-1), glypican-3 (GPC3, highly expressed in the HCC component), polyclonal carcinoembryonic antigen (pCEA, demonstrating a typical canalicular staining pattern), and cluster of differentiation 10 (CD10) [32]. To establish the cholangiocytic lineage, the preferred markers include: cytokeratin 7 (CK7), cytokeratin 19 (CK19), epithelial membrane antigen/Mucin 1 (EMA/MUC1), epithelial cell adhesion molecule (EpCAM), and cytoplasmic or membranous monoclonal CEA or monoclonal antibody 31 (MOC-31) [28]. Transitional zones frequently demonstrate co-expression of both sets of markers (e.g., co-staining of HepPar-1 and CK19 or CK7), confirming the phenotypic hybrid nature of these cells [33]. Immunohistochemical panel for cHCC-CCA is noted in Table 2.

In recent years, several specific pathological biomarkers have emerged with significant diagnostic and prognostic utility. Nestin, an intermediate filament protein and a marker of neural progenitor cells, has been identified as a highly specific and sensitive biomarker for cHCC-CCA. A clinical study by Calderaro and colleagues demonstrated that nestin immunohistochemical expression discriminates cHCC-CCA from pure HCC with an area under the curve (AUC) of 0.85, achieving a sensitivity of 75% and specificity of 93% [34]. Furthermore, high nestin expression (defined as >30% positive neoplastic cells) was found to be an independent predictor of poor disease-free survival and overall survival (OS) [34]. Additionally, delta-like homolog 1 (DLK1), a stem cell marker, is expressed in transitional and stem-cell-like areas of cHCC-CCA, and its presence in more than 5% of tumor cells is strongly associated with an aggressive clinical phenotype, early postoperative recurrence, and worse overall survival odds [16].

## 6. Molecular and Genomic Landscape

Comprehensive genomic and transcriptomic analysis has shown that cHCC-CCA is not a single molecular entity. Instead, it represents a biologically diverse spectrum that spans both hepatocellular and cholangiocellular differentiation [35].

The landmark study by Xue and colleagues (2019) utilized whole-exome and RNA sequencing on 133 cases of cHCC-CCA, demonstrating a monoclonal origin for the combined and mixed subtypes, meaning that both the HCC and iCCA components arise from a single malignant clone [36]. The authors found that TP53 mutations occurred in 49% of cases and Telomerase Reverse Transcriptase (TERT) promoter mutations in 23%, making them the most common recurrent genetic changes. These mutations are early foundational drivers present in both differentiation lineages [36].

In addition, somatic mutations in chromatin remodeling genes (ARID1A, ARID2, PBRM1, BRD7) and cellular energetics genes (KEAP1, IDH1, APOB, ALB) were frequently identified [37]. Profiling studies have shown that individual tumors often exhibit a predominant phenotype, enabling their classification as either ‘HCC-like’ or ‘CCA-like’ [37]. A clinical genomic study conducted by Murugesan and colleagues in 2021 utilized a machine learning classifier to analyze 73 cases of combined cHCC-CCA. The study classified 58% of the tumors as HCC-like, 16% as CCA-like, and 26% as ambiguous in Table 3 [37]. HCC-like cHCC-CCAs are characterized by an enrichment of TERT promoter, CTNNB1 (encoding beta-catenin, a signature of non-proliferating class HCC), and MYC alterations [37]. In contrast, CCA-like cHCC-CCAs harbor genetic alterations classic to iCCA, specifically FGFR2 fusions, IDH1 mutations, and ARID1A alterations [38]. Crucially, the study demonstrated that approximately 24.6% of all cHCC-CCA tumors harbor potentially actionable alterations, including BRCA2 mutations, ERBB2 (HER2) amplifications, MET amplifications, FGFR2 rearrangements, and IDH1 mutations [39]. These findings support the routine implementation of next-generation sequencing and molecular tumor boards for advanced cHCC-CCA [39]. Molecular subtypes and key driver alterations in cHCC-CCA are noted in Table 3.

## 7. Surgical Interventions and Curative Strategies

Surgical resection remains the primary and only well-established curative treatment option for patients with resectable cHCC-CCA, associated with significantly superior long-term survival outcomes compared to palliative or non-surgical approaches [40]. The main objective of surgical resection is to achieve an R0 resection (microscopically negative tumor margins) while preserving a sufficient future liver remnant (FLR) volume to prevent postoperative hepatic insufficiency [11]. In patients with standard liver function and adequate FLR, major hepatectomy (the resection of three or more segments) represents the preferred surgical strategy, as a resection margin greater than 10 mm has been demonstrated to correlate with significantly prolonged disease-free survival and reduced local recurrence [11]. On the other hand, in patients with underlying cirrhosis and portal hypertension, minor hepatectomy is preferred to prevent the devastating effects of post-hepatectomy liver failure, though major resection tends to confer superior oncological clearance when feasible [41].

The utility of routine regional lymphadenectomy remains a subject of surgical debate. Although hilar lymph node involvement occurs in 12–33% of cHCC-CCA cases, retrospective studies demonstrate conflicting results regarding whether systematic lymph node dissection improves overall survival versus adding surgical morbidity [42,43].

In patients with advanced liver cirrhosis where surgical resection is functionally prohibitive due to portal hypertension, liver transplantation (LT) represents a potentially transformative therapeutic option. LT simultaneously eradicates the tumor and eliminates the pro-oncogenic cirrhotic microenvironment [44]. Recent multi-center studies reveal that carefully chosen patients with early-stage cHCC-CCA can experience outstanding long-term survival following transplantation, with 5-year OS rates reaching as high as 93.3% in strictly selected cohorts meeting Milan or early-stage criteria [45]. Nevertheless, these outstanding outcomes reflect highly selective protocol enrolment in small sample sizes; broader application of LT is limited by organ donor shortages and high recurrence risks in unselected or locally advanced cases making limited evidence supporting its use in cHCC-CCA currently [46].

## 8. Locoregional and Systemic Therapeutic Options

For patients with primarily unresectable, locally advanced, or recurrent cHCC-CCA, locoregional therapies (LRT) play a critical role in disease control, symptom palliation, or as a bridging and downstaging therapy for potential curative intervention [47]. Transarterial chemoembolization (TACE) represents the most common LRT utilized for advanced disease [48]. However, the clinical response to TACE is highly dependent on tumor vascularity: globally hypervascular (HCC-dominant) tumors achieve an impressive objective response rate (ORR) of up to 85%, whereas hypovascular (peripherally vascular and centrally fibrotic, iCCA-dominant) tumors exhibit a poor response rate of only 10% [49]. Transarterial radioembolization (TARE) using yttrium-90 (Y-90) microspheres has emerged as a highly promising alternative, demonstrating partial response rates of 55% to 60% and a high disease control rate of 60% to 65%, with median OS exceeding 9 to 16 months [50]. For small tumor nodules (<=3 cm) in patients with compromised liver function, radiofrequency ablation (RFA) or microwave ablation (MWA) are effective focal therapies [51].

The absence of standardized systemic treatment guidelines represents a major therapeutic gap for advanced cHCC-CCA, as these patients have been systematically excluded from landmark phase III clinical trials in both HCC and iCCA [52]. Historically, clinicians have derived treatment regimens from either HCC or iCCA parent tumor types, depending on the prevalent radiological or serum characteristics [52]. Retrospective clinical evidence in 30 patients supports cytotoxic gemcitabine plus platinum-based chemotherapy (such as GemCis or GEMOX) as the preferred therapy, achieving median OS of 10 to 16.2 months, median progression-free survival (PFS) of 8 to 9.0 months, and ORR of 15% to 29% [53]. In contrast, the HCC-directed tyrosine kinase inhibitor (TKI) sorafenib has shown consistently poor efficacy, with a median OS of only 3.5 to 10.7 months and ORR of 0% to 10%, showing significant inferiority to platinum-containing chemotherapy in multiple multicenter cohorts of 36 patients [54]. Lenvatinib, however, shows more promising activity, achieving an ORR of 42.9% and disease control rate (DCR) of 92.9% in a small Japanese cohort of 14 patients [55]. Since most available evidence for cHCC-CCA comes from retrospective studies with limited sample sizes, further and more rigorous research is required to verify the therapeutic efficacy.

The integration of immune checkpoint inhibitors has recently transformed the systemic therapy landscape. Based on the finding that approximately 57% of cHCC-CCA tumors display an ‘immune-high’ phenotype characterized by rich T-cell recruitment and activation of cytotoxic pathways, immunotherapy is highly active in this disease [33]. The combination of atezolizumab (PD-L1 inhibitor) and bevacizumab (anti-VEGF antibody) achieved an ORR of 33% and a median OS of 13.0 months in a French multicenter retrospective study of 16 patients [56]. Furthermore, a groundbreaking real-world study by Lin and colleagues (2025) evaluated a triple combination therapy consisting of interventional treatment (TACE/Hepatic Arterial Infusion Chemotherapy), PD-(L)1 inhibitors, and targeted therapy (TKI/bevacizumab) in 51 patients with unresectable cHCC-CCA [57]. The triple therapy demonstrated outstanding clinical efficacy, yielding a median OS of 17.8 months, a median PFS of 8.9 months, and a remarkable ORR of 41.2% (by RECIST 1.1) and 56.9% (by mRECIST), with manageable toxicities [57]. This triple-modality regimen represents an emerging conversion therapy, successfully downstaging 21.6% of initially unresectable tumors for curative resection [57]. As most relevant studies involving immunotherapy are retrospective and involve small patient populations, further and more rigorous research is required to verify the therapeutic efficacy. Clinical efficacy and strategic rationale of selected treatment regimens are noted in Table 4.

The identification of potentially targetable alterations in approximately 25% of cHCC-CCA tumors emphasizes the absolute necessity of comprehensive genomic profiling for all patients with advanced disease. In the CCA-like subtype, FGFR2 fusions/rearrangements and IDH1 mutations are present in up to 25% of cases [30]. These patients are potential candidates for FGFR inhibitors like pemigatinib, futibatinib, and lirafugratinib, as well as IDH1 inhibitors such as ivosidenib. This assessment is based on encouraging trial data from biliary tract cancer studies [52]. Although evidence from conventional iCCA cannot be directly extrapolated to cHCC-CCA, with potential clinical relevance, further research is required to verify its therapeutic implications. Additionally, HER2 (ERBB2) amplification, present in a subset of tumors, can be targeted with trastuzumab deruxtecan under tumor-agnostic approvals, while rare BRCA2 mutations have demonstrated remarkable clinical responses to the PARP inhibitor olaparib [58].

## 9. Prognostic Predictors and Risk Classification

The prognosis for patients diagnosed with cHCC-CCA is generally poor. For those with advanced or recurrent disease, the median survival is around 8 months. Even after surgical resection, the 5-year survival rates vary between 10% and 30% in different clinical studies [7]. Clinicians must therefore utilize a highly precise risk-stratification system to identify high-risk patients and guide intensive follow-up protocols.

Evolving clinical data have identified several key adverse prognostic factors associated with poor patient survival and early tumor recurrence [59]. These include advanced age (>=60 years), tumor size greater than 5 cm, presence of multiple tumor nodules (multifocality), macrovascular and microvascular invasion, hilar lymph node metastasis, positive resection margins, lack of complete tumor capsule formation, and elevated pre-treatment serum markers (CA19-9, CEA, and gamma-glutamyl transferase >60 U/L) [60].

Importantly, the clinical prognosis is heavily influenced by the disease subtype and histomorphology. Under the 2024 Gurzu et al. hypothesis, ‘iCCA-type’ cHCC-CCAs, which demonstrate a prominent fibrotic desmoplastic stroma and a strong predilection for lymph node metastasis, are associated with a significantly worse prognosis than ‘HCC-type’ tumors, which mimic the clinical course of conventional HCC [1]. Furthermore, the presence of more than 5% stem/progenitor cell features and high expression of markers such as nestin or DLK1 are established independent predictors of an aggressive phenotype, tumor recurrence, and dismal survival odds [61].

Currently, there is no specific American Joint Committee on Cancer (AJCC) staging system specifically for cHCC-CCA; in the 8th Edition, it is staged similarly to iCCA, though recent studies suggest that the HCC TNM staging system provides superior prognostic stratification and survival correlation for resectable cases [62].

## 10. Conclusions and Future Directions

cHCC-CCA remains one of the most complex, aggressive, and phenotypically heterogeneous primary liver malignancies. Over the past decades, our clinical approach has transitioned from diagnostic ambiguity to a refined, histology-driven standard codified by the 2019 WHO 5th Edition classification. The integration of advanced multi-phasic imaging, multi-site core biopsies, and biomarkers like nestin has greatly reduced previous diagnostic delays and sampling errors.

Concurrently, high-throughput genomic and transcriptomic analyses have revealed the monoclonal origin of these tumors, defining them as a biological continuum primarily categorized into “HCC-like” and “CCA-like” molecular subtypes. This type of molecular classification is paving a clear path for precision oncology. Biliary tract cancer subtypes characterized by FGFR2 fusions, IDH1 mutations, or HER2 amplifications are rapidly emerging as therapeutic targets for specific small-molecule inhibitors and antibody-drug conjugates. Meanwhile, studies have found that more than half of such tumors exhibit a “high-immunity” microenvironment; this finding provides a strong rationale for employing combination therapies involving immune checkpoint inhibitors and triple-modality conversion therapy, with the aim of downstaging advanced, unresectable tumors to enable curative surgical resection.

Despite these advances, significant gaps remain in treatment. Patients with cHCC-CCA are typically excluded from landmark Phase III clinical trials—a situation that underscores the absolute necessity of establishing dedicated multicenter prospective registries and conducting clinical trials. Moving forward, the management of this “double whammy” will depend entirely on timely next-generation sequencing, dynamic molecular tumor boards, and biomarker-stratified multidisciplinary care models stratified by biomarkers—approaches designed to target the unique dual lineages driving this challenging disease.

## Figures and Tables

**Table 1:** Historical Evolution of Classification Systems for cHCC-CCA.

Classification	Key Subtypes & Diagnostic Criteria	Clinical & Molecular Relevance
**Allen & Lisa (1949) [9]**	• Type A (double): Separate nodules of HCC & iCCA • Type B (combined): Contiguous nodules with intermingling • Type C (mixed): Single mass with fine-scale intermixture	First historical classification. Type C represents the true hybrid variant and is associated with aggressive clinical course and TERT promoter mutations.
**Goodman et al. (1985) [10]**	• Type I: Collision tumors (coincidental separate nodules) • Type II: Transitional tumors (progressive transition) • Type III: Fibrolamellar tumors (resembled fibrolamellar HCC but containing mucin-producing pseudoglands)	Goodman Type II Transitional closely matches modern biological definitions. Type III Fibrolamellar arises de novo in younger patients and shows better survival.
**WHO 2010 (4th Edition) [11]**	• Classical Type: Unequivocal mixed elements • Stem Cell Subtypes: Typical, Intermediate-Cell, and Cholangiolocellular	Identified potential origin from bipotent progenitors, but faced poor diagnostic reproducibility. Cholangiolocellular and typical stem features show extremely poor prognosis.
**WHO 2019 (5th Edition) [12,13,14]**	• cHCC-CCA (definitive): Unequivocal dual H&E differentiation • Intermediate-Cell: Monotonous intermediate phenotype • Cholangiolocellular reassigned under small duct iCCA	Mandated strict morphology on H&E. Excluded collision tumors and reassigned cholangiolocelluar under iCCA, enhancing classification reproducibility and homogeneity.

Abbreviations: cHCC-CCA—combined hepatocellular-cholangiocarcinoma, HCC—hepatocellular carcinoma, iCCA—intrahepatic cholangiocarcinoma, TERT—telomerase Reverse Transcriptase, H&E—hematoxylin and eosin.

**Table 2:** Diagnostic Immunohistochemical Panel for cHCC-CCA.

Target Lineage	Primary Biomarkers	Staining Characteristics & Diagnostic Significance
**Hepatocytic**	HepPar-1, ARG-1, GPC3, pCEA, CD10	HepPar-1 and ARG-1 show high sensitivity. GPC3 is strongly positive in HCC component. pCEA shows characteristic canalicular pattern.
**Cholangiocytic**	CK7, CK19, EMA/MUC1, EpCAM, monoclonal CEA, MOC-31	Cytoplasmic staining for CK7/CK19. Monoclonal CEA and MOC-31 show cytoplasmic/membranous expression in biliary structures.

Abbreviations: cHCC-CCA—combined hepatocellular-cholangiocarcinoma, HCC—hepatocellular carcinoma, HepPar-1—hepatocyte paraffin 1, ARG-1—arginase-1, GPC3—glypican-3, pCEA—polyclonal carcinoembryonic antigen, CD10—cluster of differentiation 10, CK7—cytokeratin 7, CK19—cytokeratin 19, EMA/MUC1—epithelial membrane antigen/Mucin 1, EpCAM—epithelial cell adhesion molecule, CEA—carcinoembryonic antigen, MOC-31—monoclonal antibody 31.

**Table 3:** Molecular Subtypes and Key Driver Alterations in cHCC-CCA.

Molecular Subtype	Prevalence (%)	Key Genetic Drivers & Therapeutic Actions
**HCC-like**	58% (42/73)	Enriched in TERT promoter, CTNNB1, and MYC alterations. Often shows immune-low characteristics.
**CCA-like**	16% (12/73)	Harbors classic iCCA alterations: FGFR2 fusions, IDH1 mutations, and ARID1A alterations. Candidates for targeted FGFR/IDH1 inhibitors.
**Ambiguous**	26% (19/73)	Intermediate molecular profile, lacking clear single-lineage dominance on genomic classification.

Abbreviations: cHCC-CCA—combined hepatocellular-cholangiocarcinoma, HCC—hepatocellular carcinoma, CCA—cholangiocarcinoma, iCCA—intrahepatic cholangiocarinoma, TERT—telomerase Reverse Transcriptase, CTNNB1—a gene that is located on the short arm (p) of chromosome 3 at position 22.1, MYC—myelocytomatosis, FGFR2—fibroblast growth factor receptor 2, IDH1—isocitrate dehydrogenase 1, ARID1A—AT-rich interaction domain-containing protein 1A.

**Table 4:** Clinical Efficacy, Study Design, and Evidence Qualification of Systemic Regimens.

Intervention/Regimen	Study Design & Sample Size (*N*)	Reported Clinical Efficacy	Evidence Qualification & Rationale
**Gemcitabine + Platinum (GemCis/GEMOX)**	Multicenter Retrospective Cohorts (Salimon et al., 2017 [*N* = 30]) [53]	• Median OS: 10.2–16.2 mo • Median PFS: 8.0–9.0 mo • ORR: 15%–29%	Most widely preferred regimen based on retrospective cohort evidence. Highly effective because cHCC-CCA’s hypo-vascular and stroma-dense nature renders standard TKIs less active.
**Sorafenib (Conventional TKI)**	Multicenter Retrospective Series (Kobayashi et al., 2018 [*N* = 36]) [54]	• Median OS: 3.5–10.7 mo • Median PFS: 2.8–4.8 mo • ORR: 0%–10%	Shows consistently limited activity and significant inferiority to chemotherapy in multicenter studies, though occasionally useful in HCC-dominant cases.
**Lenvatinib (Multi-Kinase TKI)**	Small Retrospective Cohort (Tanabe et al., 2025 [*N* = 14]) [55]	• Median OS: 14.9 mo • Median PFS: 6.1 mo • ORR: 42.9% • DCR: 92.9%	Investigational/off-label option. Multi-kinase inhibition (FGFR1-4, VEGF1-3) provides dual activity against both biliary and vascular components.
**Atezolizumab + Bevacizumab (ICI + VEGF)**	Multicenter Retrospective Cohort (Gigante et al., 2023 [*N* = 16]) [56]	• Median OS: 13.0 mo • Median PFS: 3.0 mo • ORR: 33.0%	Exploratory off-label extrapolation from HCC standard. Demonstrates encouraging activity in tumors with immune-active microenvironments.
**Triple Combination (TACE/HAIC + PD-(L)1 + TKI)**	Real-World Retrospective Cohort (Lin et al., 2025 [*N* = 51]) [57]	• Median OS: 17.8 mo • Median PFS: 8.9 mo • ORR: 41.2% (RECIST)/56.9% (mRECIST)	Exploratory conversion protocol. Down-staged 21.6% of unresectable tumors for curative resection.

Abbreviations: cHCC-CCA—combined hepatocellular-cholangiocarcinoma, HCC—hepatocellular carcinoma, TKI—tyrosine Kinase Inhibitor, ICI—immune Checkpoint Inhibitor, FGFR—Fibroblast growth factor receptor, VEGF—vascular Endothelial Growth Factor, TACE—transcatheter arterial chemoembolization, HAIC—hepatic arterial infusion chemotherapy, PD-(L)1—programmed cell death-ligand 1, OS—overall survival, PFS—progression-free survival, ORR—objective response rate, DCR—disease control rate, RECIST—response evaluation criteria in solid tumors, mRECIST—modified response evaluation criteria in solid tumors, *N*—patient number.

## Data Availability

No new data were created or analyzed in this study. Data sharing is not applicable to this article.

## Abbreviations

The following abbreviations are use in this manuscript:

AFP	Alpha-fetoprotein
ARG-1	Arginase-1
ARID1A	AT-rich interaction domain-containing protein 1A
CA19-9	Carbohydrate antigen 19-9
CD10	Cluster of differentiation 10
CEA	Carcinoembryonic antigen
CEUS	Contrast enhanced ultrasound
cHCC-CCA	Combined hepatocellular–cholangiocarcinoma
CK7	Cytokeratin 7
CK19	Cytokeratin 19
CT	Computed tomography
ctDNA	Circulating tumor deoxyribonucleic acid
CTNNB1	A gene that is located on the short arm (p) of chromosome 3 at position 22.1
DCR	Disease control rate
EMA/MUC1	Epithelial membrane antigen/Mucin 1
EpCAM	Epithelial cell adhesion molecule
FGFR	Fibroblast growth factor receptor
FLR	Future liver remnant
GPC3	Glypican-3
HAIC	Hepatic arterial infusion chemotherapy
HCC	Hepatocellular carcinoma
H&E	Hematoxylin and eosin
HepPar-1	Hepatocyte paraffin 1
iCCA	Intrahepatic cholangiocarcinoma
ICG-R15	Indocyanine green retention test at 15 min
IDH1	Isocitrate dehydrogenase 1
ICI	Immune Checkpoint Inhibitor
MOC-31	Monoclonal antibody 31
*N*	Patient number
mRECIST	Modified response evaluation criteria in solid tumors
MRI	Magnetic resonance imaging
MWA	Microwave ablation
MYC	Myelocytomatosis
ORR	Objective response rate
OS	Overall survival
pCEA	Polyclonal carcinoembryonic antigen
PD-L1	Programmed cell death-ligand 1
PFS	Progression-free survival
RECIST	Response evaluation criteria in solid tumors
RFA	Radiofrequency ablation
TACE	Transcatheter arterial chemoembolization
TARE	Transarterial radioembolization
TERT	Telomerase Reverse Transcriptase
TKI	Tyrosine Kinase Inhibitor
VEGF	Vascular Endothelial Growth Factor
